# Cysteine-Induced Hybridization of 2D Molybdenum Disulfide Films for Efficient and Stable Hydrogen Evolution Reaction

**DOI:** 10.3390/ma14051165

**Published:** 2021-03-02

**Authors:** Arunas Jagminas, Paulius Gaigalas, Carla Bittencourt, Vaclovas Klimas

**Affiliations:** 1 The Department of Electrochemical Materials Science, State Research Institute Center for Physical Sciences and Technology, Sauletekio ave. 3, LT-01257 Vilnius, Lithuania; paulius.gaigalas@ftmc.lt (P.G.); vaclovas.klimas@ftmc.lt (V.K.); 2 Chemie des Interactions Plasma-Surface, University of Mons, Place du Parc 22, 7000 Mons, Belgium; Carla.BITTENCOURT@umons.ac.be

**Keywords:** molybdenum disulfide, L-cysteine, hydrothermal synthesis, hybrid films, electrocatalyst, water splitting

## Abstract

The noble, metal-free materials capable of efficiently catalyzing water splitting reactions currently hold a great deal of promise. In this study, we reported the structure and electrochemical performance of new MoS_2_-based material synthesized with L-cysteine. For this, a facile one-pot hydrothermal process was developed and an array of densely packed nanoplatelet-shaped hybrid species directly on a conductive substrate were obtained. The crucial role of L-cysteine was determined by numerous methods on the structure and composition of the synthesized material and its activity and stability for hydrogen evolution reaction (HER) from the acidic water. A low Tafel slope of 32.6 mV dec^−1^, close to a Pt cathode, was registered for the first time. The unique HER performance at the surface of this hybrid material in comparison with recently reported MoS_2_-based electrocatalysts was attributed to the formation of more defective 1T, 2H-MoS_2_/MoO_x_, C nanostructures with the dominant 1T-MoS_2_ phase and thermally degraded cysteine residues entrapped. Numerous stacks of metallic (1T-MoS_2_ and MoO_2_) and semiconducting (2H-MoS_2_ and MoO_3_) fragments relayed the formation of highly active layered nanosheets possessing a low hydrogen adsorption free energy and much greater durability, whereas intercalated cysteine fragments had a low Tafel slope of the HER reaction. X-ray photoelectron spectroscopy, scanning electron microscopy, thermography with mass spectrometry, high-resolution transmission electron microscopy, Raman spectroscopy techniques, and linear sweep voltammetry were applied to verify our findings.

## 1. Introduction

Hydrogen gas produced from water electrolysis via catalytic splitting is ascribed to a mostly clean energy carrier. However, to achieve relevance for practical usage, the hydrogen evolution reaction (HER) rate, as at the surface of a Pt-based electrode, which exhibits the best HER performance [1], requires alternative cheaper catalysts.

Over the past decade, numerous reports have been devoted to the synthesis of various nanostructured materials for catalysis of water splitting reactions. Among them, nanoscale MoS_2_ species have been the most intensively investigated 2D material because of the specific graphene-like layered morphology and unique catalytic, biological, and energy-related properties [2,3,4,5]. However, the intrinsic conductivity, catalytic activity, and stability of the pure and the most thermodynamically sTable 2H-MoS_2_ nanostructured films are usually poor in comparison with Pt group metals and compounds [6]. In addition, the overvoltage of pristine 2H-MoS_2_ nanoplatelets for HER is significantly larger, about −0.2 V vs. reference hydrogen electrode, RHE, potential [7,8,9,10] compared with Pt/C [7]. Therefore, much effort has been devoted to the development of novel, more effective hybrid electrocatalysts. In this context, MoS_2_ films doped by other elements [11]; hybridized with 1T-MoS_2_ [12,13], WO_3_ [14], and glycine [15]; decorated with various guest nanoparticles [16,17,18]; deposited onto graphite [19], graphene, and graphene oxides [20,21]; and carbon nanotubes [22] have been proposed. Various methods have been explored to synthesize layered MoS_2_ nanomaterials, including laser ablation [23], thermal decomposition [24], gas-phase reaction [25], magnetron sputtering [26], and hydrothermal [27] or sonochemical [28] processing. An efficient strategy to enhance HER activity of MoS_2_-based electrocatalysts is to design highly conducive substrates, such as 3D-structured graphene [29,30], graphene oxide [31], foams [32,33], carbon fiber [34], etc. [22]. Various HER reaction Tafel slopes from 41 to 68 mV dec^−1^ and overpotentials from −185 to −260 mV were reported for the best samples. As is known, better HER performance is characteristic for the metasTable 1T (1T’) phase compared with 2H-MoS_2_ atoms and the transformation to the sTable 2H-MoS_2_ phase. It is crucial to synthesize sTable 1T (1T’)-MoS2-based catalysts [35,36].

It has been both experimentally and theoretically proven that the catalytically active sites for HER are located just at the unsaturated sulfur atoms of the MoS_2_ nanoplatelet edges because the edge sites in 2D MoS_2_ have near-zero hydrogen absorption free energy (0.08 eV) [37,38]. Consequently, the engineering of defect-rich MoS_2_ nanostructures directly influence the activity of MoS_2_-based electrocatalysts the most. Therefore, various post-treatment methods resulting in the design of highly-defective MoS_2_ through etching [39], doping [40,41], intercalation [42], and nanoparticulation [36] have been reported. However, the hybridization of MoS_2_ nanostructures with cysteine amino acid resulting in the formation of an HER electrocatalyst with a surprisingly low Tafel slope of 32.6 mV dec^−1^, which is the lowest among the reported for MoS_2_ materials, has not yet been reported.

Here, we reported the synthesis recipe, chemical composition, structure, and electrochemical performance of a new hybrid material composed of the dominant 1T-MoS_2_ phase heterostructured with 2H-MoS_2_, Mo(IV), and Mo(VI) oxide fragments and carbon. We showed that the engineered hybrids have enhanced electrochemical performance and significantly higher stability in comparison with pure 2D MoS_2_, as exhibited by an increase in the HER current density to over 80 mA cm^−2^ at −0.35 V overvoltage.

The samples were characterized by means of X-ray photoelectron spectroscopy (XPS), X-ray diffraction (XRD), scanning electron microscopy (SEM), high-resolution transmission electron microscopy (HRTEM), thermogravimetry (TG), and differential thermal spectroscopy (DTA) coupled with mass spectrometry, Raman spectroscopy, and cycling voltammetry.

## 2. Results and Discussion

**Figure 1:**
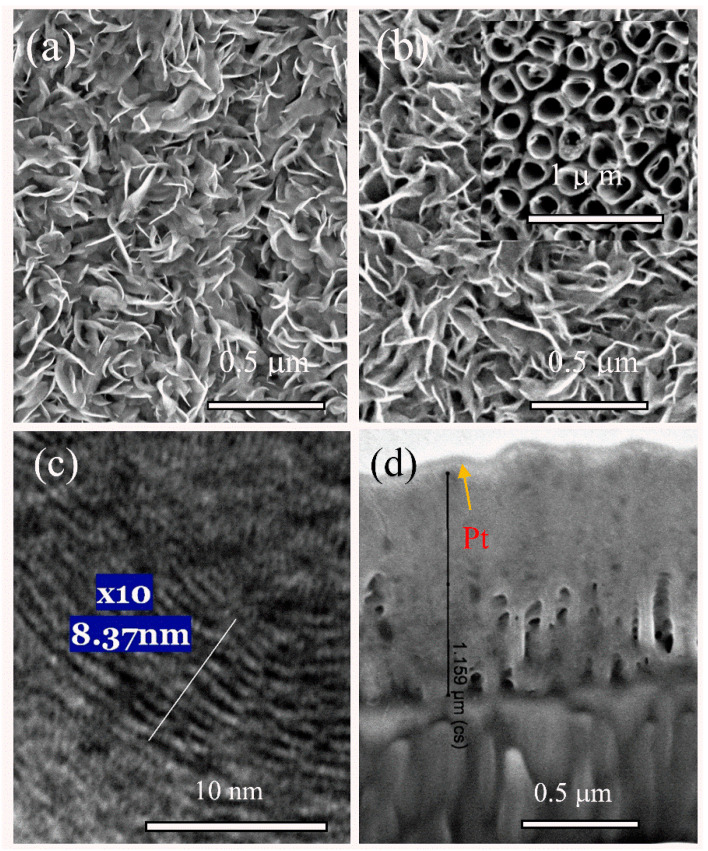
Top-side (**a**,**b**) and cross-sectional (**d**) scanning electron microscopy (SEM) and high-resolution transmission electron microscopy (HRTEM) (**c**) images of film synthesized hydrothermally on the natotubed titania surface (inset) from the solution containing 5.0 (NH_4_)_6_Mo_7_O_24_ 4H_2_O, 90 thiourea without (**a**) and containing 2.0 mmo L^−1^ L-cysteine (**b**–**d**) at 220 °C for 5 h.

Hydrothermal processing of the ammonium heptamolybdate and the thiourea solution at 220–225 °C resulted in the formation of crystalline MoS_2_ nanoplatelet species in the solution bulk [15]. In this way, uniform and well-attached nanoplatelet films can also be engineered directly at various substrates. The thickness, varying from 0.7 to 2.5 μm, of these black-colored films is mainly dependent on the autoclaving time [15]. The morphology of films formed under the same autoclaving conditions in the solution without and containing L-cysteine was quite similar (Figure 1a).

The HRTEM image (Figure 1b) clearly revealed that the atomic flatness of the sandwiched layers in the composite MoS_2_-cyst film was greatly disrupted because of a non-periodic atom arrangement and increased to 8.4 Å distance between S-Mo-S lattices compared with the characteristic one of crystalline molybdenite 2H-MoS_2_ (6.15 Å) [43]. However, a markedly larger variation in the Mo-to-Mo spacing, nonlinear atom distributions, and the presence of numerous twists can be viewed. In many sites, the spacing between neighboring monolayers exceeded 10 Å. According to previous reports, significantly larger distances between the stacked S-Mo-S planes compared with characteristic of pure molybdenite were found, which implied the intercalation of guest molecules.

**Figure 2:**
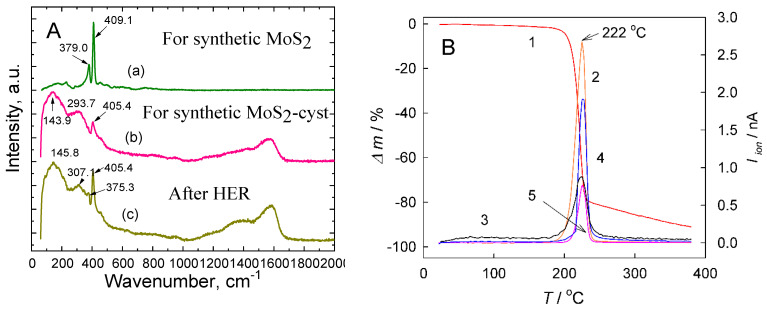
(**A**) Raman spectra of (a) as-grown MoS_2_ and (b) MoS_2_-cys films at the Ti/TiO_2_ substrate as well as (c) the same MoS_2_-cys film after the HER processing by potential cycling within a range of 0.05 to −0.35 V vs. the RHE potential for 1000 scans; (**B**) (1) thermogravimetry (TG) plot and variables of (2) CO_2_, (3) H_2_O, (4) NH_3_, and (5) H_2_S ionic currents during annealing of L-cysteine in argon determined by mass spectrometry (MS) analysis.

To further study the intercalation of cysteine or thiourea molecules or fragments inside the film, Raman spectroscopy investigations were performed. Figure 2a displays the Raman spectra of the films designed at the Ti/TiO_2_ substrate by hydrothermal synthesis in the basic.

Solution without (a) and with 2 mmol L^−1^ L-cysteine (b,c) before (a,b) and after (c) the prolonged HER processing. As for film synthesized without cysteine, two clearly resolved peaks at 409.1 cm^−1^ and 379.0 cm^−1^, attributable to the A_1g_ and E_2g_^1^ longitudinal acoustic phonon modes, respectively, and typical for the crystalline 2H-MoS_2_ [20] from few-layered flakes, were detected. The Raman spectrum of the as-grown MoS_2_-cyst film is shown in Figure 2a,b. This spectrum apart of a low intensity A_1g_ mode peaked at 405.4 cm^−1^. The broad additional vibration modes peaked at 143.9 and 293.7 cm^−1^ and the very broad mode peaked in the 1100–1650 cm^−1^ region. These modes can be associated with the presence of molybdenum oxides and organic molecule fragments, respectively, entrapped inside this film [44].

A similar shape of the Raman spectrum is also characteristic for MoS_2_-cys film after the prolonged usage as an HER catalyst by 1000 potential scans within a 0.05 to −0.35 V window (Figure 2a). However, for this film, a significantly sharper and stronger A_1g_ mode was determined, likely indicating the presence of a larger amount of crystalline MoS_2_ phase compared with the as-grown film.

Identification of gaseous species with *m*/*z* = 44 (CO_2_), *m*/*z* = 17 (NH_3_), and *m*/*z* = 34 (H_2_S) released during the thermal decomposition of cysteine in an argon atmosphere via evolved gas analytical mass spectrometry revealed that all functional groups, namely -COOH, -SH, and -NH_2_ ought to be detached from the cysteine molecule at around 220 °C (Figure 2b). Therefore, it was difficult to suspect the intercalation or adsorption of cysteine molecules inside and onto the 2D MoS_2_ nanoflakes. This allowed us to draw the important conclusion that an increased HER performance at the hybrid MoS_2_-cyst electrocatalyst cannot be related to adsorption and intercalation of cysteine molecules; from the DTA/MS analysis of all emitted species as well as the XPS data, it could have been COS or CS_2_ species formed via the thermal splitting of thiourea and cysteine in a synthesis reactor.

**Figure 3:**
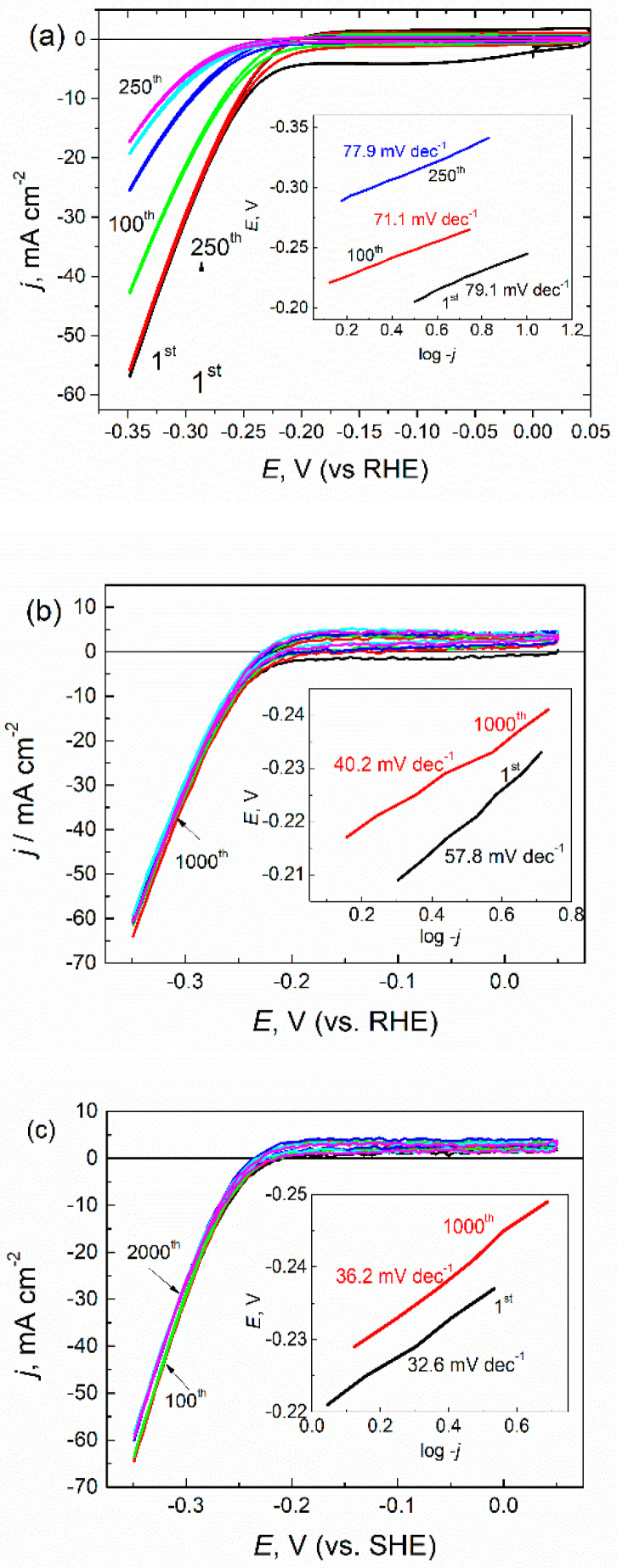
The sets of cyclic voltammograms recorded in the H_2_-saturated solution of 0.5 H_2_SO_4_ mol L^−1^ at a 10 mV s^−1^ potential sweep rate of: (**a**) pure MoS_2_ film and the same film hybridized with (**b**) 1.0 and (**c**) 3.0 mmol L^−1^ of L-cysteine. In the insets are the Tafel slopes calculated for the indicated specimen and the potential scan cycle.

To evaluate the electrolytic HER activity, we performed linear sweep voltammetry (LSV) measurements in a typical three-electrode setup. To assess the durability of the hybrid electrocatalyst, up to 2000 potential sweeps were conducted. Figure 3 shows the sets of LSV curves obtained.

At a potential scan rate of 10 mV s^−1^ in argon-saturated 0.5 mol L^−1^ H_2_SO_4_ solution (pH = 0), films were synthesized without (a) and with L-cysteine (b,c). From these, the onset potential of HER at the cysteine-free as well as the cysteine-used MoS_2_ electrode approximated to about −0.2 V vs. RHE. Additionally, from the current density variables, only some higher activity within all tested potential window were obtained for electrodes fabricated using L-cysteine. However, the electrochemical performance of HER at the electrodes grown in the L-cysteine-containing reactor differed significantly. The potential cycling within the 0.05 to −0.35 V potentials window of the L-cysteine-free electrode usually resulted in an HER-activity decrease down to 15–18 mA cm^−2^ just after 250 cycles (Figure 3a), implying about a 70% decay from initial activity. In contrast, the electrodes covered with nanoplatelet-shaped hybrid MoS_2_/cysteine film showed considerable higher HER activity in the same potentials window, decreasing insignificantly during the subsequent 2000 potential scans (Figure 3b,c). A further benefit is that MoS_2_/cysteine hybrid HER electrocatalyst possessed significantly lower Tafel slopes (Figure 3b,c) compared with the ones synthesized under the same conditions without cysteine. In the case of films synthesized in the presence of just 1 mmol L^−1^ cysteine, the prolonged HER processing resulted in the marked decrease in the Tafel slope value from 57.8 to 40.2 mV dec^−1^, ca. by 30% (Figure 3b). An increase in the cysteine concentration to 3 mmol L^−1^ resulted in the surprisingly low Tafel slope value (32.6 mV dec^−1^) of as-grown film, which was the lowest among all reported data for MoS_2_-based electrocatalysts and was close to the Tafel slope value characteristic for the HER at the Pt and Pt/C substrates. The Tafel slope value of 30 mV dec^−1^ indicates that the recombination reaction H_ads_ + H_ads_ → H_2_ is an HER rate-limiting stage followed by a fast discharge reaction: H_3_O^+^ + e → H_ads_ + H_2_O, whereas the Tafel slopes of 40 mV dec^−1^ indicates that H_2_ evolution proceeds via a Volmer–Heyrovsky pathway [45].

**Figure 4:**
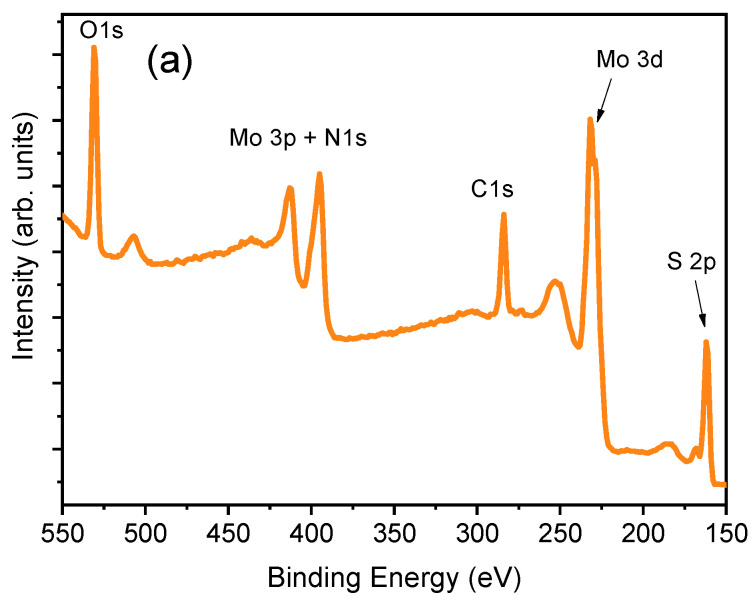

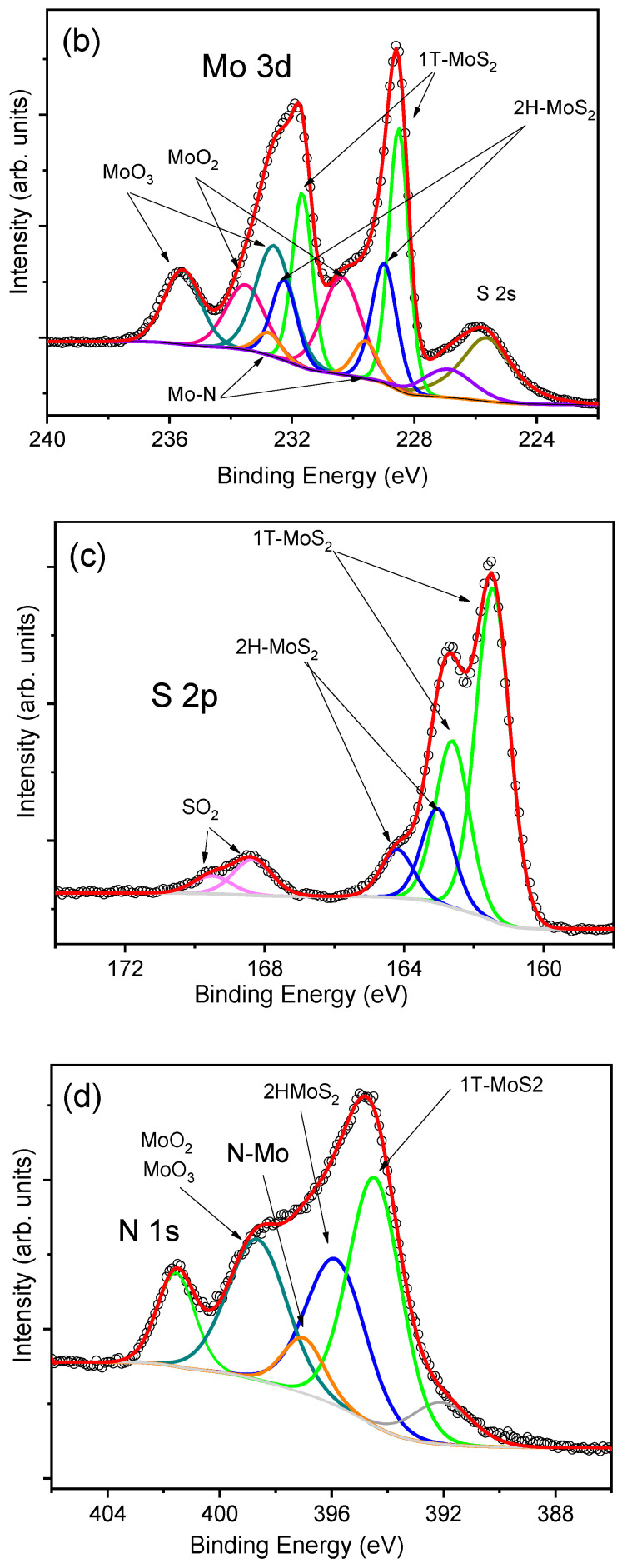

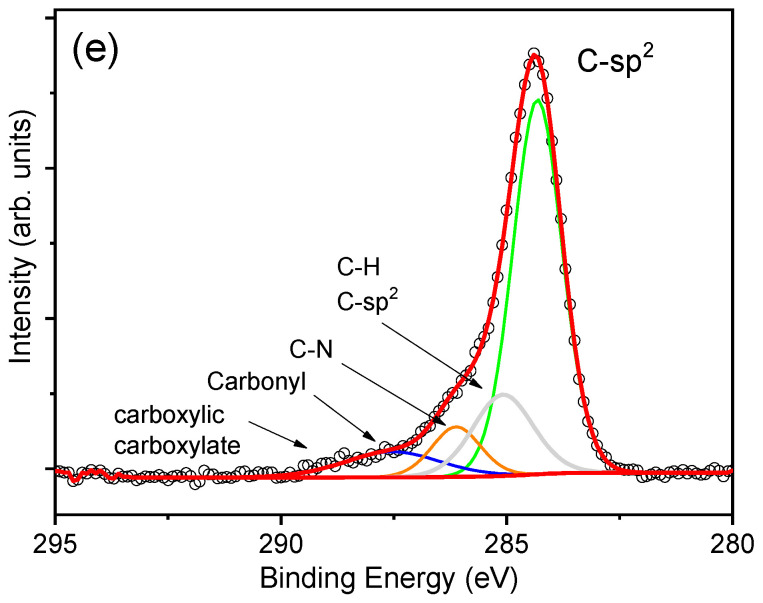
X-ray photoelectron spectroscopy (XPS) spectra of as-grown MoS_2_-cyst film: (**a**): survey, (**b**): Mo3d, (**c**): S 2p, (**d**): Mo 3p), and (**e**): C1s.

X-ray photoelectron spectroscopy was used to investigate the chemical states of the elements in the surface region of the synthesized films. The full-range survey spectrum taken from the MoS_2_-cyst sample is shown in Figure 4a, from which the presence of the Mo, S, O, N, and C elements was evidenced. 

Figure 4b revealed the presence of Mo^4+^ in both the semiconducting 2H-MoS_2_ and the metallic 1T-MoS_2_ and MoO_2_ phonon modes, whose 3d_5/2_ and 3d_3/2_ binding energies (BEs) were 229.0 and 232.2 eV for the 2H phase [46], 228.5 and 231.7 eV for 1T-MoS_2_ [13], and 230.4 and 233.6 eV for MoO_2_ [13,47], respectively, with the most intensive peaks for the thermodynamically metasTable 1T-MoO_2_. A similar conclusion can be drawn from the analysis of S 2p BE peaks presented in Figure 4c. For this spectrum, the doublets with components at 161.5 and 162.6 eV correspond to the BEs of S 2p_3/2_ and 2p_1/2_ states in the 2H-MoS_2_, whereas the most intense peaks are characteristic of the octahedral 1T phase. It is worth noticing that some amounts of Mo^6+^ and S^4+^ were also determined, for which the binding energies of 232.5 eV and 235.6 eV (Mo 3d_5/2_ and Mo 3d_3/2_, respectively), were attributed to MoO_3_, and 168.4 and 169.6 eV (S 2p_3/2_ and S 2p_1/2_, respectively) to SO_2_ [48]. The analysis of Mo 3p and C 1s modes also revealed the presence of nitrogen and carbon (Figure 4d,) in the hybrid film synthesized with L-cysteine, indicating the formation of multiphasic 1T/2H-MoS_2_-MoO_x_/C,N material. With regard to the N 1s spectrum, the peak located at 401.6 eV should be ascribed to the intercalation of NH_4_^+^, whereas the BE peaked at 397.0 eV can be attributed to the formation of Mo-N bond. From the XPS analysis, the concentration of elements was C, Mo, S, O, and N equaled to 32.8 at.% for C, 11.5 at.% for Mo, 25.3 at.% for S, 26.4 at.% for O, and 4.0 at.% for N.

The interactions between various amino acids (AA) and single-layer MoS_2_ nanosheet have been theoretically investigated by Dong et al. [49], concluding that no chemical bonds formed between them. The adsorption strength of AA on MoS_2_ depended on the AA type influencing the spatial distribution of the HOMO and LUMO orbitals and the MoS_2_ band gap decreased. As reported by Dong et al., the cysteine adsorption at the single layer 2H-MoS_2_ substrate reduced the band gap by 0.27 eV because of the enhanced hybridization between the Mo d-orbital and the S p-orbital after oxygen incorporation [49]. Therefore, it is reasonable to suggest that adsorption of cysteine at the nanosheets surface of the hybrid multiple-layered MoS_2_ film could modulate its catalytic properties towards becoming more active and stable. However, we did not determine the insertion of cysteine molecules, although the XP spectra revealed insertion of C and N atoms inside the MoS_2_-cyst products. Since cysteine molecules start degrading at around 200 °C, the formation and insertion of MoS_2_ fragments at 220 °C may be expected. The insertion of quest species, as shown in the study, was also elucidated by the greatly disrupted S-Mo-S layers and the increased distance between two neighboring ones (see Figure 1c). We suggest that insertion of quest species may affect the catalytic stability and activity of 1T-/2H-MoS_2_/MoO_x_ hybrid films because of the formation of a higher amount of catalytically active sites and the stabilization of the highly conducting 1T-MoS_2_ phase. The low Tafel slope determined for this HER electrocatalyst (32.6 mV dec^−1^), which is much smaller than that of the bulk MoS_2c_, could be explained as follows.

According to Weiss et al. [50], the thermal degradation of cysteine molecules proceeds via this reaction:

2Cyst = C_6_H_14_O_4_S_2_ → 2CO_2_ + 2H_2_S + NH_3_ + C_4_H_7_N (1)

The pathway to the formation of C_4_H_7_N could be the ejection of the carboxyl group, –C*OOH, and the –SH group from Cyst. The remaining chain NH_2_–C_α_–C* is short, but still suitable for intercalation, further cyclization, and formation of 2,5-dihydro-1H-pyrole (ChemSpider 13870958) with 69 Da or another pyroline with the double bond elsewhere in the ring. The exact composition of the intercalated species has not been elucidated yet. However, if intercalated cysteine fragments are like NH_2_–C_α_–C*, the processing of HER at the MoS_2_/NH_2_–C_α_–C* edges may be changed significantly towards a reaction with a low Tafel slope, as established in this study. Further studies are currently being carried out by Furje-transformed infrared (FTIR) spectroscopy and nuclear magnetic resonance (NMR).

## 3. Conclusions

Here, we report on the one-pot hydrothermal synthesis of the hybrid-type 2D MoS_2_-based electrocatalyst for efficient hydrogen evolution from the acidic solution. Based on the XPS, SEM, HRTEM, and TG results it was inferred that the increased activity and stability of a novel hybrid MoS_2_ film are related to the formation of a 2D composite from dominating metallic-type and highly active 1T-MoS_2_ and MoO_2_ phases interfaced with the semiconducting 2H-MoS_2_ and MoO_3_ phases and carbon. The surprisingly low Tafel slope of 32.6 mV dec^−1^ for this HER electrocatalyst in the strongly acidic aqueous solution was determined for the first time implying that H_2_ formation at this electrode proceeds via the Volmer–Tafel pathway. This was attributed to the possible insertion between two neighboring S–Mo–S nanosheets of thermally degraded cysteine residue species, like NH_2_–C_α_–C*. These findings highlight the need to study further the influence of amino acid on the formation mechanism of hybrid films.

## 4. Materials and Methods

### 4.1. Materials and Chemicals

Ammonium heptamolybdate tetrahydrate (NH_4_)_6_Mo_7_O_24_ 4H_2_O (99.5%) was obtained from Reachem (Bratislava, Slovakia), whereas thiourea, (NH_2_)_2_CS (99%), and L-cysteine were purchased from Sigma-Aldrich (St. Louis, MO, USA) and used as received. The Ti specimens with the working surface of 1.0 cm^2^ (7 × 7 mm^2^) and a tag (1 × 30 mm^2^) were cut from Ti foil (99.7 at%, 0.127 mm thick, Aldrich). For Ti surface anodizing, NH_4_F and H_3_PO_4_ were purchased from Reachem, Bratislava, Svovakia. Aqueous solution for hydrogen evolution was prepared from deionized water (18.4 MΩ) and analytically grade sulfuric acid.

### 4.2. Ti Surface Preparation and Anodizing

The surface of samples was ultrasonically cleaned in acetone, ethanol, and water (6 min in each) and air dried. Ti samples anodizing was conducted in the thermostated Teflon cell containing 2.0 mol L^−1^ H_3_PO_4_ and 0.5 mol L^−1^ of NH_4_F at 17 ± 0.3 °C and 20 V for 1 h. Two platinum plates were used as cathodes. After anodizing, the specimens were thoroughly rinsed, air-dried, and calcined at 450 °C for 2 h using a 10 °C min^−1^ ramp. An anatase TiO_2_ nanotubed sublayer between Ti and MoS_2_ was chosen as the substrate for the nanostructured HER catalyst because of its fairy good chemical and thermal stability, huge surface area, low resistance in the hydrogen environment, and because of the adsorption affinity of the MoS_2_ species to titanium oxide [51,52].

### 4.3. Synthesis

To cover the Ti/TiO_2_ samples with catalytically active nanostructured MoS_2_ film, the hydrothermal processing was conducted in a Teflon line stainless steel autoclave (25 mL in volume) at 220 °C for 5 to 10 h using a 10 °C min^−1^ ramp. An aqueous solution of 5 mmol L^−1^ ammonium heptamolybdate and 90 mmol L^−1^ thiourea without and containing up to 5 mmol L^−1^ of L-cysteine was used. The synthesized products were collected by centrifugation, rinsed thoroughly, and dried at 60 °C. To obtain densely packed MoS_2_ nanoplatelet films well-attached to the substrate, the Ti specimen covered with the nanotubed anatase TiO_2_ layer in a thickness of about 1 μm was inserted inside the reactor.

### 4.4. Raman Spectra

Raman investigations were performed on an inVia (Renishaw, New Mills, UK) spectrometer equipped with a thermoelectrically cooled (−70 °C) CCD camera. Spectra were excited at 532 nm by a diode-pumped solid-state laser on a ~2 μm diameter spot with power at the sample = 0.06 mW. The accumulation time was 400 s. Raman scattering wavenumber axis was calibrated by the silicon peak at 520.7 nm. To determine the parameters of the bands, the fitting of experimental spectra with the Gaussian–Lorentzian shape components using GRAMS/A1 8.0 (Thermo Scientific) software (version 8.0, Thermo Electron Corp.). was conducted.

### 4.5. SEM and HRTEM

The morphology and microstructure of the films and species obtained were analyzed using a scanning electron microscope (FEI Helios Nanolab 650, Eindhoven, The Netherlands) and a high-resolution transmission microscope FEI TECNAI F20 (Eindhoven, The Netherlands). To estimate their elemental composition, the products were analysed with a CrossBeam Auriga Workstation (Eindhoven, The Netherlands) equipped with a field emission gun and an energy dispersive *X*-ray spectrometer.

### 4.6. XPS

X-ray photoelectron spectroscopy (XPS) was used to evaluate the relative elemental composition of the samples. For the XPS measurements, a spectroscope VERSAPROBE PHI 500 from Physical Electronics (Physical Electronics, Chanhassen, MN, USA) was used; the excitation source was a monochromatized AlK. The energy resolution was 0.6 eV. A dual-beam charge neutralization (electron gun (~1 eV) and Argon Ion gun (<10 eV)) was used for charge compensation. The C1s peak at 284.3 eV was used for binding energy calibration.

### 4.7. TG/HDSC-MS

A simultaneous thermal analysis STA Pt 1600 (Linseis, Germany) apparatus equipped with a mass spectrometer MS Thermostar GDS 320 (Linseis/Pfeiffer, Germany) was used for research on the thermal decomposition processes of L-cysteine. For this, the specimen of 10 mg weight in PtRh cans was evacuated and then heated at 10 °C min^−1^ in an argon atmosphere. The data were collected and fitted using the Evaluation and Quadera software (version 4.62, INFICON AG, Bad Ragaz, Switzerland).

### 4.8. Cyclic Voltammetry

Electrochemical measurements were performed in a three-electrode-configured setup using a Zahner Zennium (Kronach, Germany) electrochemical workstation. The (Ag/AgCl,KCl_sat_) electrode was used as a reference, while a glassy carbon stripe with an area of ~10 cm^−2^ and Ti/TiO_2_/MoS_2_ were used as the counter and the working electrode, respectively. A linear sweep voltammetry with a scan rate of 10 mV s^−1^ within 0.05 to −0.35 V vs. the RHE potential range was conducted in 0.5 mol L^−1^ of H_2_SO_4_ pre-purged with H_2_ for 30 min. In the stability tests, up to 2000 cycles were recorded. All potentials in the text refer to RHE.

## Data Availability

Not applicable.
